# EHMTI-0074. Excessive daytime sleepiness in migraineurs is associated with anxiety and depression: a population-based study

**DOI:** 10.1186/1129-2377-15-S1-D9

**Published:** 2014-09-18

**Authors:** M Chu, BK Kim, JW Park, JM Kim

**Affiliations:** 1 Neurology, Hallym University Sacred Heart Hospital, Anyang, Korea; 2 Neurology, Eulji University School of Medicine, Seoul, Korea; 3 Neurology, The Catholic University of Korea, Uijeongbu, Korea; 4 Neurology, Chungnam National University, Daejeon, Korea

## Introduction

Excessive daytime sleepiness is a major clinical and health concern that can have harmful consequences and has shown an associated with anxiety and depression. A close relationship between EDS and migraine has been reported in case-control studies. Case-control study may be affected by confounding factors.

## Aims

To examine whether excessive daytime sleepiness (EDS) in migraineurs is associated with anxiety and depression in a population-based sample.

## Methods

We selected a stratified random population sample of Koreans aged 19-69 and evaluated them with a 60-item semi-structured interview designed to identify headache type, anxiety, depression and EDS. Subjects with EDS was identified if a subject's Epworth Sleepiness Scale (ESS) was 10 or more. Anxiety and depression symptoms were evaluated using Goldberg Anxiety Scale questions and Patient Health Questionnnaire-9, respectively.

## Results

The 1-year prevalences of EDS and migraine were 16.8% and 5.4%, respectively. Migraineurs reported more commonly reported EDS compared to non-migraine subjects (25.2% vs. 16.3%, p=0.005). Migraineurs with EDS reported higher attack frequency per month (6.0±8.5 vs. 3.5±5.8, p=0.010), higher HIT-6 score (60.0 ±10.1 vs. 52.6±8.3, p<0.001) compared to migraineurs without EDS. Logistic regression analysis revealed that migraine showed an odds ratio (OR) for EDS compared to non-migraineurs (OR [CI]) = 1.7 [1.2-2.6]). After adjusting anxiety and depression, migraine was not associated with EDS (OR [CI]) = 1.3 [0.8-1.9]).

## Conclusions

Approximately 1/4 of migraineurs experienced EDS. Excessive daytime sleepiness in migraineurs was associated with anxiety and depression.

No conflict of interest.

